# *Prevotella histicola*, A Human Gut Commensal, Is as Potent as COPAXONE® in an Animal Model of Multiple Sclerosis

**DOI:** 10.3389/fimmu.2019.00462

**Published:** 2019-03-22

**Authors:** Shailesh K. Shahi, Samantha N. Freedman, Alexandra C. Murra, Kasra Zarei, Ramakrishna Sompallae, Katherine N. Gibson-Corley, Nitin J. Karandikar, Joseph A. Murray, Ashutosh K. Mangalam

**Affiliations:** ^1^Department of Pathology, University of Iowa, Iowa City, IA, United States; ^2^Graduate Program in Immunology, University of Iowa, Iowa City, IA, United States; ^3^Medical Scientist Training Program, University of Iowa, Iowa City, IA, United States; ^4^Graduate Program in Molecular Cell Biology, University of Iowa, Iowa City, IA, United States; ^5^Department of Immunology, Mayo Clinic, Rochester, MN, United States; ^6^Division of Gastroenterology and Hepatology, Mayo Clinic, Rochester, MN, United States

**Keywords:** multiple sclerosis, gut microbiome, Copaxone®, animal model, experimental autoimmune encephalomyelitis (EAE), HLA transgenic mice, *Prevotella histicola*, immune response

## Abstract

Multiple sclerosis (MS) is a demyelinating disease of the central nervous system. We and others have shown that there is enrichment or depletion of some gut bacteria in MS patients compared to healthy controls (HC), suggesting an important role of the gut bacteria in disease pathogenesis. Thus, specific gut bacteria that are lower in abundance in MS patients could be used as a potential treatment option for this disease. In particular, we and others have shown that MS patients have a lower abundance of *Prevotella* compared to HC, whereas the abundance of *Prevotella* is increased in patients that receive disease-modifying therapies such as Copaxone® (Glatiramer acetate-GA). This inverse correlation between the severity of MS disease and the abundance of *Prevotella* suggests its potential for use as a therapeutic option to treat MS. Notably we have previously identified a specific strain*, Prevotella histicola* (*P. histicola*), that suppresses disease in the animal model of MS, experimental autoimmune encephalomyelitis (EAE) compared with sham treatment. In the present study we analyzed whether the disease suppressing effects of *P. histicola* synergize with those of the disease-modifying drug Copaxone® to more effectively suppress disease compared to either treatment alone. Treatment with *P. histicola* was as effective in suppressing disease as treatment with Copaxone®, whereas the combination of *P. histicola* plus Copaxone® was not more effective than either individual treatment. *P. histicola*-treated mice had an increased frequency and number of CD4^+^FoxP3^+^ regulatory T cells in periphery as well as gut and a decreased frequency of pro-inflammatory IFN-γ and IL17-producing CD4 T cells in the CNS, suggesting *P. histicola* suppresses disease by boosting anti-inflammatory immune responses and inhibiting pro-inflammatory immune responses. In conclusion, our study indicates that the human gut commensal *P. histicola* can suppress disease as efficiently as Copaxone® and may provide an alternative treatment option for MS patients.

## Introduction

Multiple sclerosis (MS) is an autoimmune disease of the central nervous system (CNS) and is characterized by the CNS infiltration of inflammatory cells that results in demyelination, axonal damage, and progressive neurologic disability. Collective evidence suggests that MS is caused by the destruction of the myelin sheath by aberrant T cell-mediated immune responses (1), however the etiology and pathogenesis of MS are unknown.

The interaction of both genetic and environmental factors likely plays an important role in MS pathogenesis. Genetic factors account for ~30% of disease risk as determined from studies of identical twins (2), and among these, human leukocyte antigen (HLA) class II genes show the strongest association with disease (3). In addition, environmental factors account for 70% of disease risk (4, 5), however, how these are linked with a predisposition to, or protection from, MS is unknown. Various factors such as smoking, low vitamin D levels due to insufficient exposure to sunlight, and Epstein-Barr virus infection had been linked with a predisposition to MS. Recently, we and others have shown that dysbiosis of the gut occurs in MS patients more frequently than healthy controls (HC), indicating the gut microbiota is a potential environmental factor that contributes to the etiopathogenesis of MS (6–11). Further, MS patients have a lower abundance of the gut bacteria, *Prevotella*, compared to HC and the levels of *Prevotella* are increased in patients that receive disease-modifying therapies such as Copaxone® or Interferon beta (IFN-β) (7–9, 12). The preclinical HLA-DR3.DQ8 transgenic mouse model, which expresses the human class II genes HLA-DR3 and DQ8, develops classical experimental autoimmune encephalomyelitis (EAE) disease characterized by severe brain and spinal cord pathology and can be used to study human MS (13). To determine the significance of *Prevotella* in MS, we isolated and identified a particular strain of *Prevotella*, i.e., *Prevotella histicola (P. histicola)*, and used the HLA-DR3.DQ8 transgenic mouse model of MS to demonstrate that *P. histicola* could suppress proteolipid protein (PLP)_91−110_-induced EAE (14). Thus, gut bacteria can play an important role in MS pathogenesis and certain gut bacteria showing lower abundance in MS patients can be used as potential treatment option.

Copaxone® (Glatiramer acetate-GA) is an analog of myelin basic protein that is comprised of a heterogeneous mixture of polypeptides containing the four amino acids (L- glutamic acid, L-alanine, L-lysine, and L-tyrosine), and is used as a first-line disease-modifying therapy (DMTs) for the treatment of MS. Copaxone® is thought to act by suppressing antigen-specific T cell responses in the CNS (15) and by inducing the production of protective Th2 cytokines (16, 17). However, Copaxone® alone is not always efficacious in suppressing the inflammatory response in MS patients (18, 19). Therefore, there is a need to develop additional therapeutic options that can either be used alone or in combination with Copaxone® to improve treatments for MS.

Based on our data that *P. histicola* can suppress disease in a preclinical model of MS, we hypothesized that treatment with the combination of *P. histicola* and Copaxone® would have an additive effect on disease severity. In this present study, we examined the effects of the combination therapy of *P. histicola* and Copaxone® in the HLA-DR3.DQ8 transgenice mouse model of MS. In HLA-DR3.DQ8 transgenic mice, treatment with *P. histicola* suppressed EAE as efficiently as Copaxone®, whereas the combination of *P. histicola* and Copaxone® was not more effective than either treatment alone. Administration of *P. histicola*, either alone or in combination with Copaxone®, resulted in higher frequency of CD4^+^Foxp3^+^ regulatory T cells and decreased frequency of CD4 T cells that produced pro-inflammatory cytokines. Therefore, our study demonstrates that the combination of *P. histicola* and Copaxone® does not have a synergistic effect on the treatment of MS, but that *P. histicola* is as effective as Copaxone® in suppressing disease in a preclinical mouse model of MS. The disease suppression is achieved through modulation of both regulatory CD4 T cells and those producing pro-inflammatory cytokines.

## Materials and Methods

### Mice

HLA-DR3.DQ8 double transgenic (DQ8 [DQA1^*^0103, DQB1^*^0302]-DR3 [DRB1^*^0301]) mice on the B6 background have been characterized previously (13, 20). These mice lack endogenous murine major histocompatibility complex (MHC) class II genes (AE^−/−^) and express HLA-DRA1^*^0101, DRB1^*^0301, and DQA1^*^0103, DQB1^*^0302, as described previously (13, 20). These mice will be referred as HLA-DR3.DQ8 transgenic mice throughout the text. Both male and female mice (8–12 weeks of age) were utilized in this study. Mice were bred and maintained in the University of Iowa animal facility in accordance with NIH and institutional guidelines. All experiments were approved by the Institutional Animal Care and Use Committee at the University of Iowa.

### Isolation and Culture of *Prevotella histicola*

Isolation and characterization of *P. histicola* has been described previously (14). Briefly*, P. histicola* was grown at 37°C for 3 days in 5 ml of trypticase soy broth (TSB) (Hardy Diagnostics Santa Maria, USA) in an anaerobic jar with an AnaeroPack® system (Mitsubishi Gas Chemical America). The identification of *P. histicola* was confirmed by 16SrRNA-specific PCR as described previously (14).

### Disease Induction And Scoring

HLA-DR3.DQ8 transgenic mice (8–12 weeks old) were immunized subcutaneously in both flanks with 25 μg of PLP_91−110_ that was emulsified in Complete Freund's Adjuvant–CFA containing *Mycobacterium tuberculosis* H37Ra (400 μg/mouse; Becton, Dickinson and Company, Sparks, MD, USA). Pertussis toxin (PTX) (Sigma Chemicals, St. Louis, MO; 100 ng) was administered i.v. at days 0 and 2 post immunization. C57BL/6 mice were immunized subcutaneously in both flanks with MOG_35−55_ CFA/PTX as described earlier (21). Mice were observed daily for clinical symptoms and the following scoring system was used as described previously (14): 0 for normal; 1 for loss of tail tone; 2 for hind limb weakness; 3 for hind limb paralysis; 4 for hind limb paralysis and forelimb paralysis or weakness; and 5 for morbidity/death.

### Treatment of HLA-DR3.DQ8 Transgenic Mice With *Prevotella histicola* and Copaxone®

We used two protocols for Copaxone® (Copaxone, GA, Teva Neuroscience) treatment: prophylactic (Copaxone® treatment given 10 days before disease induction) and therapeutic [Copaxone® treatment given during disease induction phase (7 days post EAE induction) and after onset of disease (12 days post EAE induction)].

In a prophylactic setting, HLA-DR3.DQ8 transgenic mice were divided into five groups (*P. histicola* alone, Copaxone® alone, *P. histicola* plus Copaxone®, PBS alone, and media alone). Copaxone® alone, *P. histicola* plus Copaxone® received 2 mg of Copaxone® (21) in 200 μl of incomplete Freund's adjuvant (IFA) (Becton, Dickinson and Company, Sparks, MD, USA) 10 days before EAE induction. PBS alone group received 200 μl of a PBS/IFA emulsion 10 days before EAE induction. EAE was induced by immunization with PLP_91−110_/CFA emulsion and 7 days post EAE induction *P. histicola* alone and *P. histicola* plus Copaxone® combination group of mice were orally gavaged with live *P. histicola* (10^8^ CFUs) every other day for a total of seven doses. Mice in the control group (media only) were orally gavaged with TSB media every other day for a total of seven doses.

Effect of *P. histicola* and Copaxone® were also tested in C57BL/6 mice in a prophylactic setting. Animals were divided into four groups and treated with *P. histicola* alone, Copaxone® alone, *P. histicola* plus Copaxone® or media only. *P. histicola* alone group were orally gavaged with 10^8^ CFUs of live *P. histicola* every other day for a total of seven doses; Copaxone® alone group were treated subcutaneously with 100 μg of Copaxone® every other day for a total of seven doses (22); whereas combination group received both *P. histicola* and Copaxone® on alternate days as in Copaxone® or *P. histicola* only groups. Mice in the control group received 200 μl of TSB media alone by oral gavage. EAE was induced by immunization with MOG_35−55_ CFA/PTX as mentioned earlier (21).

For the therapeutic protocol, we treated mice either in disease induction phase (at disease onset) or chronic disease phase (when mice develop a score of 2). As HLA-DR3.DQ8 transgenic mice develop disease around day 7 (13), for treatment during disease induction phase, mice received 1st dose of *P. histicola* at day 7 postimmunization. In second protocol, treatment was started at day 12 post EAE induction, when mice developed a disease score of 2. Mice were divided into four groups (*P. histicola* alone, Copaxone® alone, *P. histicola* plus Copaxone®, and media alone) and treated on alternate day with *P. histicola*, Copaxone®, *P. histicola* plus Copaxone® or media as described above. All mice were evaluated for clinical EAE scores for the duration of the experiment.

### Pathology

Brain and spinal cord from mice treated with *P. histicola* alone, Copaxone® alone, a combination of both *P. histicola* plus Copaxone®, or TSB media alone were histologically analyzed for inflammation and demyelination as described previously (13, 23). Briefly, mice from treated and control groups were perfused with 50 ml of Trump's fixative (0.5% glutaraldehyde + 4% paraformaldehyde) via intracardiac puncture. Brain and spinal cord were surgically removed and fixed in 10 % neutral buffered formalin (10% BFA) for 24–48 h. Spinal cord were cut into 1 mm coronal blocks, embedded in paraffin and routine processed. The resulting slide was stained with Hemotoxylin and Eosin and analyzed for pathology by a board-certified veterinary pathologist in the cortex, corpus callosum, hippocampus, brainstem, straitum, and cerebellum regions as described previously (13, 23).

### Flow Cytometry

Mononuclear infiltrating cells from the brain and spinal cord were isolated using a percoll density gradient separation method as described previously (24). Mice in each treatment group were stained with antibodies to detect surface expression of CD4 (GK1.5) and CD25 (PC61) (BD Biosciences, Franklin Lakes, NJ), whereas intracellular expression of FoxP3 and IL10 were stained using an anti-Mouse/Rat FoxP3 (FJK-16s) IL10 (FES5-16E3) staining kit (eBiosciences, San Diego, CA). Intracellular staining for IL17 and IFNγ were performed using the intracellular fixation permeabilization kit and anti-mouse IL17 and IFNγ specific antibodies from eBioscience™. Cells were also stained with antibodies to detect surface expression of CD45 (30-F11) and CD4 (clone GK1.5) to gate on the leukocyte population. Gut- associated lymphoid cells were isolated and stained with antibodies as per method described previously (25).

### Microbiome Analysis

Mouse fecal pellets were collected from different groups pre and postimmunization and post treatment. Microbial DNA extraction, 16S amplicon (V3-V5 region), and sequencing were done as described previously (14). R1 and R2 fastq reads were merged using Paired-End reAd merger (PEAR) (26), merged reads were converted to fasta and merged fasta sequences were process by Cloud Virtual Resource (CloVR) (27) to form operational taxonomic units (OTUs) at 97% similarity. PLS-DA score plots and histograms plots were generated using METAGENassist (28).

### Statistical Analysis

Differences in the frequency of regulatory T cells or cytokine-producing CD4 T cells among mice that received treatment with *P. histicola* alone, Copaxone® alone, a combination of *P. histicola* and Copaxone®, or TSB media alone were assessed by Mann-Whitney *U*-test (Table 1). Average clinical EAE scores and cumulative EAE scores were compared using 2way ANOVA with multiple comparisons of the means and non-parametric Mann-Whitney *U*-test, respectively. Statistical analyses were done with GraphPad Prism 7 (GraphPad Software, La Jolla, CA). A value of *p* ≤ 0.05 was considered significant.

**Table 1:** Effect of Copaxone® alone, *P. histicola* alone, and combination of *P. histicola* and Copaxone® on PLP_91−110_-induced EAE in HLA-DR3.DQ8 transgenic mice.

**Treatment**	**Disease incidence (%)**	**Mean onset of disease ±SD**	**Mean EAE Score ±SD**	**Number of mice with maximum severity score**					
				**0**	**1**	**2**	**3**	**4**	**5**
PBS	09/09 (100%)	10.66 ± 1.30	38.57 ± 5. 37	–	–	–	–	6	3
Medium	14/14 (100%)	10.23 ± 1.37	40.07 ± 9.02	–	–	–	–	9	5
*P. histicola*	12/16 (75%)	12.71 ± 1.92	9.78 ± 2.09	4	7	1	4	–	–
Copaxone®	10/11 (88 %)	12.40 ± 1.39	16.22 ± 8.39	1	4	4	2	–	–
Copaxone® + *P. histicola*	10/10 (100%)	13.40 ± 1.50	17.79 ± 6.70	–	4	3	3	–	–

## Results

### *P. histicola* Suppresses EAE in HLA-DR3.DQ8 Transgenic Mice as Efficiently as Copaxone®

We have shown that HLA-DR3.DQ8 transgenic mice develop severe EAE with significant brain and spinal cord pathology (13) and that EAE could be suppressed by *P. histicola* in these mice (14). In the present study, we examined whether *P. histicola* and Copaxone® can work in an additive manner to ameliorate disease in a preclinical model of MS to garner evidence for the use of this combined therapy in MS patients. We studied the effect of Copaxone® in prophylactic and therapeutic setting in combination *P. histicola* treatment in disease induction phase and after EAE induction.

**Figure 1:**
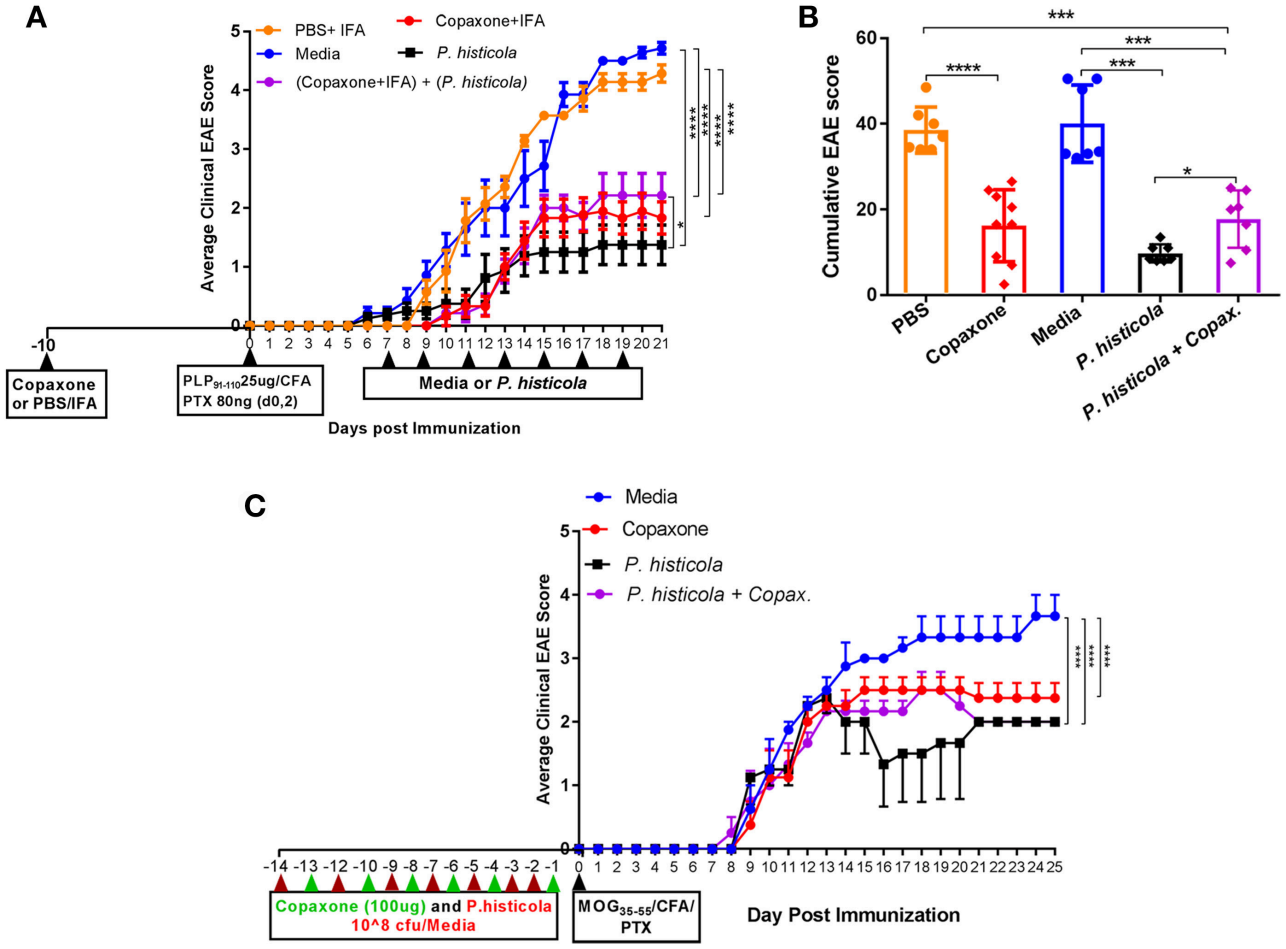
In a prophylactic setting *P. histicola* suppressed EAE similar to Copaxone® in HLA-DR3.DQ8 transgenic and C57BL/6 mice **(A)** In a prophylactic setting, HLA-DR3.DQ8 transgenic mice were divided into five groups (*P. histicola* alone, Copaxone® alone, *P. histicola* plus Copaxone®, PBS alone, and media alone). Copaxone® alone, *P. histicola* plus Copaxone® received 2 mg of Copaxone® in 200 μl of IFA 10 days before EAE induction. PBS alone group received 200 μl of a PBS/IFA emulsion 10 days before EAE induction. EAE was induced by immunization with PLP_91−110_/CFA (pertussis toxin (PTX) was administered on day of EAE induction and 2 days post-EAE induction) and 7 days post EAE induction *P. histicola* alone and *P. histicola* plus Copaxone® combination group of mice were orally gavaged with live *P. histicola* (10^8^ CFUs) every other day for a total of seven doses. Mice in the control group (media only) were orally gavaged with TSB media every other day for a total of seven doses. Clinical scores were assessed daily for the duration of the experiment. **(B)** Cumulative EAE scores of mice treated as in **(A)**. The data presented represent 1 of 3 experiments performed at different time points (*n* ≥ 7 mice per group). **(C)** C57BL/6 mice were treated with Copaxone®, *P. histicola*, or a combination of both starting day 14 prior to immunization with MOG_35−55_ CFA/PTX (with treatment administered on alternate days for a total of 14 doses, 7 doses of Copaxone® and 7 doses of *P. histicola*). Clinical scores were assessed daily for the duration of the experiment. A *p ≤ 0.05, ***p ≤ 0.0005 and ****p ≤ 0.00005 when compared to the PBS medium treated group. 2-way ANOVA Dunnett's multiple comparison test were used to calculate *p*-value among different treatment groups for average daily clinical score **(A)** and Mann-Whitney unpaired *U*-test were used to calculate *p-*value in cumulative EAE score **(B)**.

In a prophylactic setting, mice treated with Copaxone® alone had a lower average daily clinical score compared to those treated with PBS/IFA (Figure 1A). Copaxone® treated mice showed a lower cumulative EAE score compared to the PBS/IFA control group. Similarly, HLA-DR3.DQ8 transgenic mice treated with *P. histicola* showed milder disease compared to those treated with media. *P. histicola-*treated mice also had a lower cumulative EAE score compared to media-treated mice (Figure 1B and Table 1). The combination of prophylactic treatment with Copaxone® and therapeutic treatment with *P. histicola* showed similar disease suppression as treatment with Copaxone® (Figures 1A,B, and Table 1). Interestingly, *P. histicola* alone group showed better disease suppression compared to combination of Copaxone® (prophylactic treatment) and *P. histicola* (therapeutic treatment).

To determine whether *P. histicola* can suppress disease in strains other than HLA transgenic mice, we investigated disease suppressive effect of *P. histicola* in C57BL/6 mice. Prophylactic treatment with *P. histicola* suppressed disease in C57BL/6 mice similar to HLA-DR3.DQ8 transgenic mice (Figure 1C). Additionally, Copaxone® alone and Copaxone® plus *P. histicola* treated group showed milder disease and lower average daily clinical score compared to those treated with media only (Figure 1C). Thus, our data indicate that treatment with *P. histicola* alone is as effective in suppressing disease as treatment with Copaxone® in HLA-DR3.DQ8 transgenic mice as well as C57BL/6 mice. Additionally, the combination of *P. histicola* plus Copaxone® is not more effective than either treatment alone.

**Figure 2:**
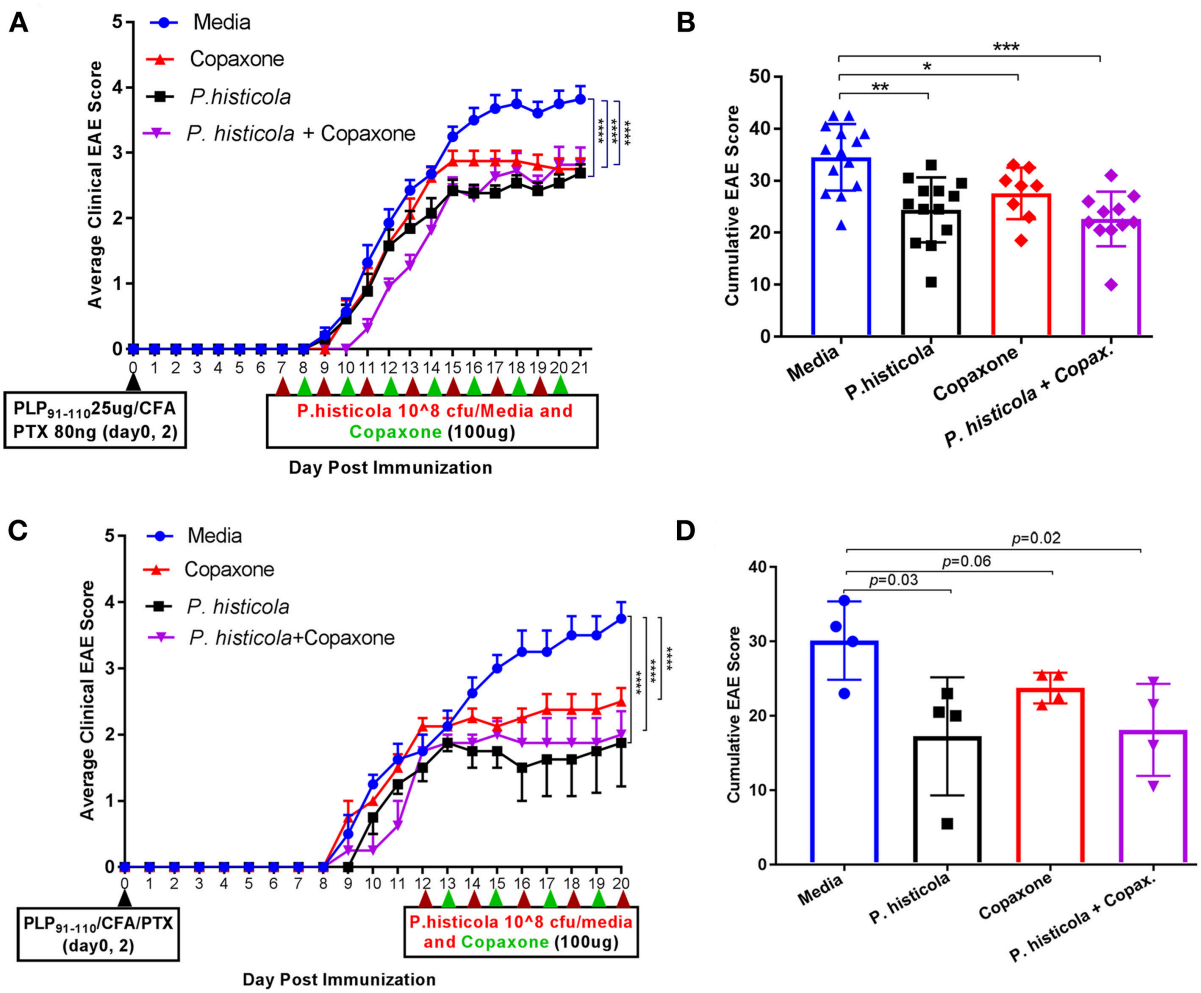
In therapeutic setting *P. histicola* suppresses PLP_91−110_-Induced EAE in HLA-DR3.DQ8 transgenic mice similar to therapeutic treatment of Copaxone®. **(A)** Mice were immunized with PLP_91−110_/CFA plus pertussis toxin on days 0 and 2 of the disease induction and 1 week later mice were treated with Copaxone®, *P. histicola* or a combination of both (with treatment administered on alternate days for a total of 14 doses, 7 doses of Copaxone® and 7 doses of *P. histicola*) for 2 weeks. Clinical scores were assessed daily for the duration of the experiment. **(B)** Cumulative EAE scores of mice treated as in **(A)**. **(C)** Mice were treated with Copaxone®, *P. histicola* or a combination of both after 12 days of immunization. Clinical scores were assessed daily for the duration of the experiment. **(D)** Cumulative EAE scores of mice treated as in **(C)**. The data presented represent 1 of 3 experiments performed at different time points (*n* ≥8 mice per group). A *p ≤ 0.05, **p ≤ 0.005, ***p ≤ 0.0005 and ****p ≤ 0.00005 when compared to the medium treated group. 2way ANOVA Dunnett's multiple comparisons test were used to calculate *p*-value in average clinical EAE score **(A,C)** and Mann–Whitney unpaired *U*-test were used to calculate *p-*value in cumulative EAE score **(B,D)**.

In the prophylactic setting described above, mice received Copaxone® 2 weeks prior to start of treatment with *P. histicola*. Thus, it could be argued that Copaxone® had a dominant effect in suppressing disease because it was given prior to the start of the treatment with *P. histicola*. Therefore, we asked whether there was a synergistic effect when treatment with both Copaxone® and *P. histicola* was started at the same time. We used two therapeutic protocols with mice receiving first treatment at either day 7 postimmunization (disease induction phase) or day 12 postimmunization when mice develop a disease score of 2 (post disease development). Since in prophylactic setting media and PBS alone group showed similar effect therefore we excluded PBS alone group in therapeutic setting. Treatment with Copaxone® alone or *P. histicola* alone resulted in a lower average daily clinical score (Figure 2A) and the cumulative EAE score compared to treatment with media alone (Figure 2B). Mice that received a combination of both *P. histicola* and Copaxone® showed a slight delay in disease onset, but had a similar average daily clinical score (Figure 2A) and cumulative average EAE score (Figure 2B) compared to the groups receiving *P. histicola* or Copaxone® alone. In second protocol, HLA-DR3.DQ8 transgenic mice received first treatment when majority of mice develop score of 2 (12 days following EAE induction). Copaxone® alone, *P. histicola* alone and Copaxone® plus *P. histicola* treated group showed milder disease compared to the control media only group (Figures 2C,D). Thus, our data indicate that *P. histicola* is as effective at suppressing EAE when administered alone or in combination with Copaxone®, and is similar to treatment with Copaxone® alone in both preventive and therapeutic settings.

### Treatment With *P. histicola* or Copaxone® Reduces Inflammation and Demyelination in The CNS

**Figure 3:**
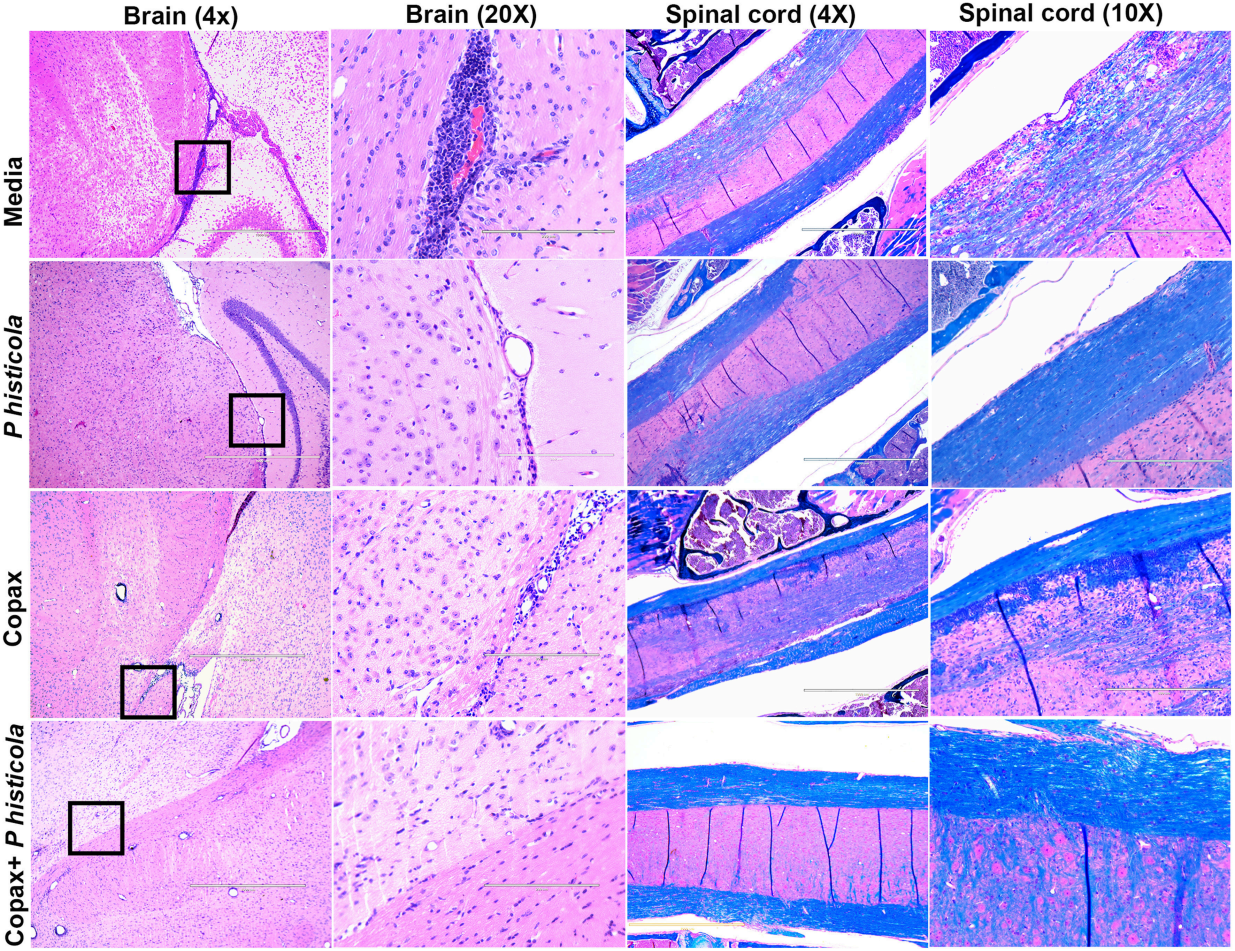
Treatment with *P. histicola* alone, Copaxone® alone, or the combination of *P. histicola* and Copaxone® resulted in decreased inflammation and demyelination in the brain and spinal cord of HLA-DR3.DQ8 transgenic mice induced with EAE. Representative hematoxylin and eosin (H&E)-stained images of the brain or Luxol-fast blue stained images of spinal cords of mice treated with *P. histicola* alone, Copaxone® alone, *P. histicola* and Copaxone®, or media. 20X Brain section (inset black boxes in 4X) show regions of inflammation. Similarly spinal cord sections are enlarged at 10X to show regions with inflammation and demyelination. Data are representative of 3 independent experiments (*n* = 5 mice per group).

To determine whether disease suppression upon treatment with *P. histicola*, Copaxone®, or the combination of both was associated with milder CNS pathology, we analyzed CNS tissues from mice induced with EAE that received treatment with either *P. histicola* alone, Copaxone® alone, the combination of both treatments, or media. Mice that were treated with *P. histicola* alone, or the combination of both treatments showed lower inflammation and demyelination in the brain and spinal cord compared to mice treated with media alone (Figure 3). Brain sections from mice treated with media showed higher number of inflammatory cells in the meningeal and stratum region (Figure 3). Copaxone® alone treated group showed few inflammatory regions in the brain, however the inflammation was milder than media only group. In contrast mice treated with *P. histicola* alone, Copaxone® alone, or the combination of *P. histicola* and Copaxone® showed mild meningeal inflammation (Figure 3). Similarly, spinal cord sections from mice that received media showed severe demyelination (i.e., loss of myelin sheath) and inflammation, whereas spinal cord sections from mice treated with *P. histicola* alone, Copaxone® alone, or the combination of *P. histicola* plus Copaxone® showed only mild inflammation in a few small areas without any significant demyelination (Figure 3). Thus, treatment with *P. histicola* alone, Copaxone® alone, or the combination of *P. histicola* plus Copaxone® can reduce CNS pathology in mice induced with EAE.

### *P. histicola* Induces CD4^+^FoxP3^+^ Regulatory T cells in HLA-DR3.DQ8 Transgenic Mice

**Figure 4:**
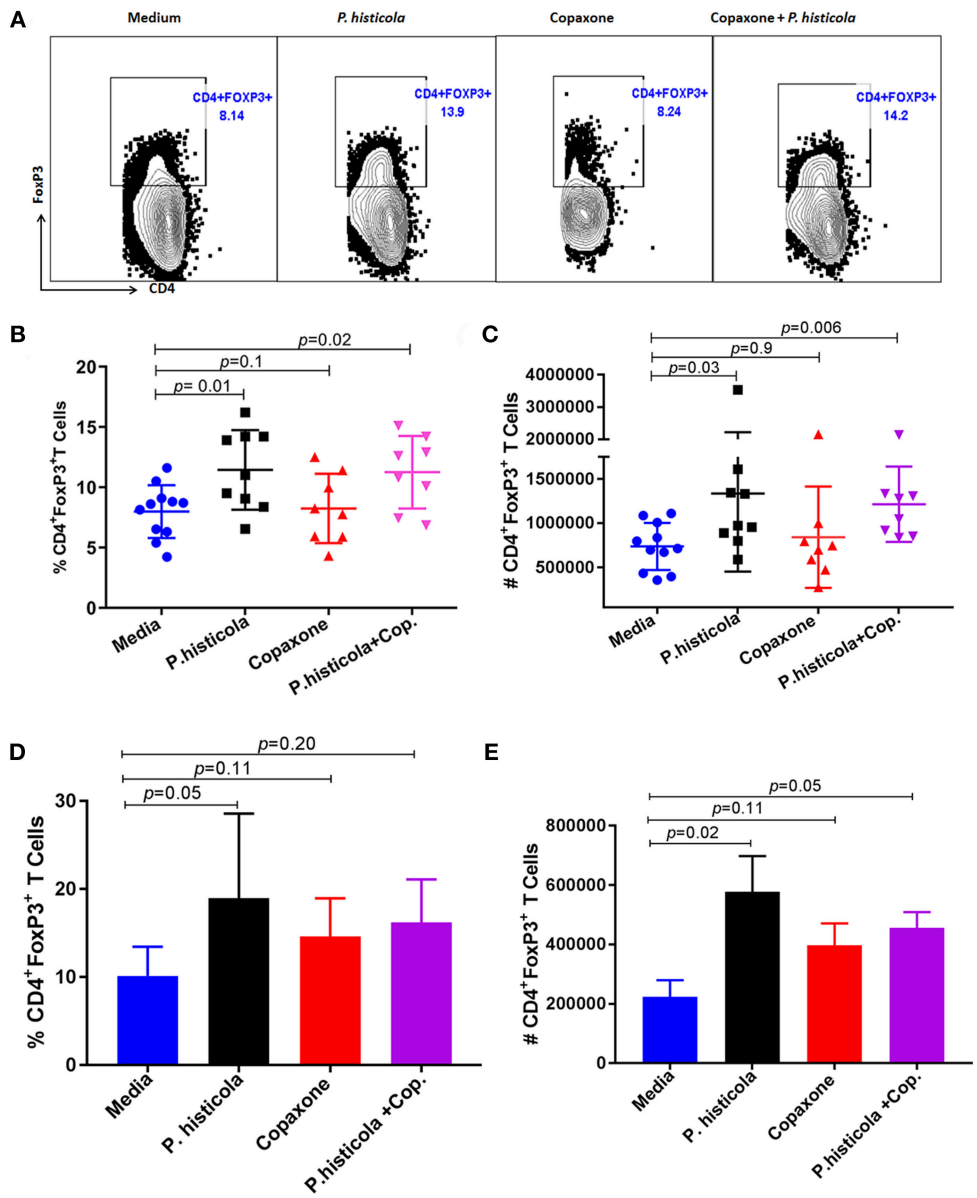
Treatment with *P. histicola* alone or in combination with Copaxone® increases CD4^+^FoxP3^+^ regulatory T cells in the spleen and GALT. **(A)** Mice were immunized with PLP_91−110_/CFA PTX on days 0 and 2 of the disease induction and 1 week later mice were treated with Copaxone® (7 doses), *P. histicola (7 doses)*, or a combination of both (with treatment administered on alternate days for a total of 14 doses, 7 doses of Copaxone® and 7 doses of *P. histicola*). Clinical scores were assessed daily for the duration of the experiment. Representative flow cytometric plots to demonstrate CD4^+^FoxP3^+^ regulatory T cells in the spleen of mice treated with *P. histicola* alone, Copaxone® alone, *P. histicola* and Copaxone®, or media. Cells were previously gated on lymphocytes and singlets. **(B)** Frequency of CD4^+^FoxP3^+^ regulatory T cells from mice treated as in **(A)**. **(C)** Quantification of the number of CD4^+^FoxP3^+^ regulatory T cells in mice treated as in **(A)**. **(D)** Naïve mice were treated with Copaxone® (7 doses), *P. histicola* (7 doses), or a combination of both (with treatment administered on alternate days for a total of 14 doses). Gut-associated lymphoid cells were isolated from treated and control group of mice and stained with CD45, CD4 and FoxP3 antibodies. Frequency of CD4^+^FoxP3^+^ regulatory T cells from mice treated Copaxone®, *P. histicola, P. histicola* plus Copaxone®, and media. **(E)** Quantification of the number of CD4^+^FoxP3^+^ regulatory T cells in mice treated as in **(D)**. Error bars are presented as standard error of the mean. The *p*-value determined by Mann–Whitney unpaired *U*-test for comparing each group to media. The data presented represent 1 of 3 experiments performed at different time points (*n* ≥ 7 mice per group).

CD4^+^FoxP3^+^ regulatory T cells play an important role in suppressing EAE disease (29). Therefore, we analyzed whether CD4^+^FoxP3^+^ regulatory T cells were associated with disease suppression upon treatment with *P. histicola*, Copaxone®, or the combination of both *P. histicola* and Copaxone®. Splenocytes from mice that were induced with EAE by immunization with PLP_91−110_ and received treatment with *P. histicola* alone, Copaxone® alone, the combination of *P. histicola* plus Copaxone®, or media alone were stained for CD4^+^FoxP3^+^ regulatory T cells. Treatment with *P. histicola* alone (*P. histicola* vs. media: 11.44 ± 3.29 vs. 7.99 ± 2.19, *p* > 0.01) or the combination of *P. histicola* and Copaxone® (*P. histicola* plus Copaxone® combination vs. media: 11.25 ± 3.00 vs. 7.99 ± 2.19 vs. *p* < 0.01) resulted in a higher frequency and number of CD4^+^FoxP3^+^ regulatory T cells compared to mice treated with media alone (*P. histicola* vs. media: 1336270 ± 883218 vs. 737246 ± 265858, *P* < 0.01, *P. histicola* plus Copaxone® combination vs. media: 1213568 ± 425699 vs. 737246 ± 265858, *p* > 0.001) (Figures 4A–C). Notably, we did not observe an increased frequency of CD4^+^FoxP3^+^ regulatory T cells in mice that only received Copaxone® treatment (Figures 4A–C). Since, *P. histicola* is given by oral gavage, it can mediate its effect through modulation of immune cells in the gut, therefore we analyzed levels of CD4^+^FoxP3^+^ regulatory T cells and CD4^+^ IL-10 cells in gut-associated lymphoid tissue (GALT). *P. histicola* alone treated group showed higher number of CD4^+^FoxP3^+^ regulatory T cells compared to media treated group (Figures 4D,E). Combination of *P. histicola plus* Copaxone® treated group only showed higher number but not frequency of CD4^+^FoxP3^+^ regulatory T cells. We could not detect any measurable level of IL-10 producing CD4+ T cells in nay groups (data not shown). Copaxone® alone group did not show any difference in CD4^+^FoxP3^+^ regulatory T cells (Figures 4D,E). Thus, *P. histicola* alone or in combination with Copaxone® modulates disease by inducing an anti-inflammatory immune response that is mediated by CD4^+^FoxP3^+^ regulatory T cells in gut as well as periphery. Further, our data suggests that *P. histicola* and Copaxone® might utilize different regulatory pathways to suppress disease as treatment with Copaxone® failed to increase either the frequency or number of CD4^+^FoxP3^+^ regulatory T cells in either gut or periphery (Figure 4).

### Treatment With *P. histicola* Alone or in Combination With Copaxone® Reduces Antigen- Specific Th1 and Th17 Cytokines in the CNS of Mice Induced With EAE

**Figure 5:**
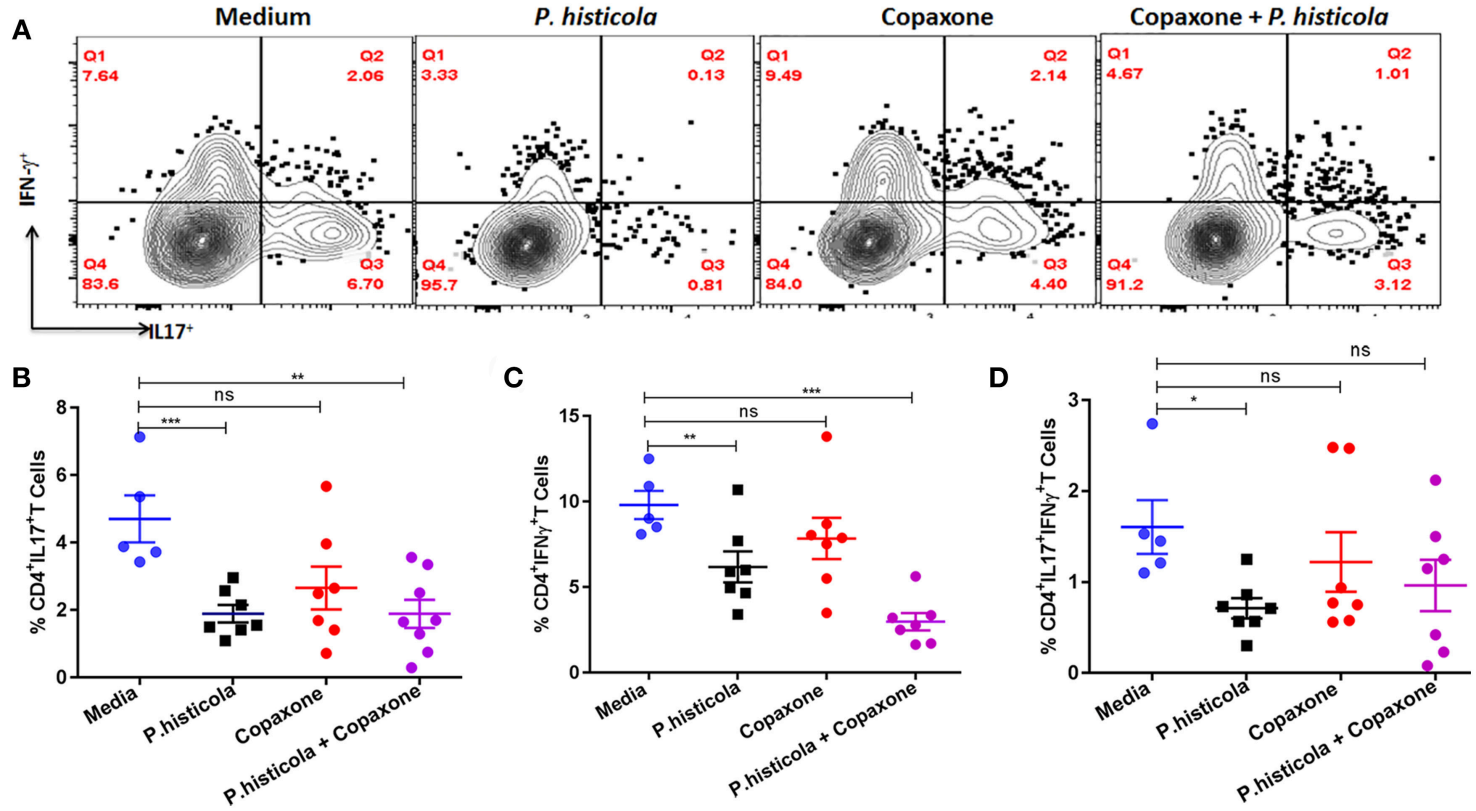
HLA-DR3.DQ8 transgenic mice induced with EAE and treated with *P. histicola* alone or in combination with Copaxone® show a decrease in inflammatory cytokine-producing cells in the CNS. **(A)** Mice were immunized with PLP_91−110_/CFA plus pertussis toxin on days 0 and 2 of the disease induction and 1 week later mice were treated with Copaxone® (7 doses), *P. histicola (7 doses)*, or a combination of both (with treatment administered on alternate days for a total of 14 doses, 7 doses of Copaxone® and 7 doses of *P. histicola*). Clinical scores were assessed daily for the duration of the experiment. Flow cytometric plots of IL17^+^- or IFNγ^+^-expressing mononuclear cells that were isolated from the brain and spinal cord of mice treated with *P. histicola* alone, Copaxone® alone, *P. histicola* and Copaxone®, or media. Cells were isolated and stimulated with antigen (PLP_91−110_) plus Brefeldin A for 12 h. Plots were previously gated on CD4^+^ cells. **(B–D)** Quantification of the frequency of CD4^+^IL17^+^T cells **(B)**, CD4^+^IFNγ^+^ T cells **(C)**, and CD4^+^IL17^+^IFNγ^+^ T cells **(D)** from mice treated as in **(A)**. Cells were previously gated on lymphocytes, singlets, and CD4^+^ cells. The data presented are the average of 2 independent experiments with *n* = 4 mice per group. The *p*-value determined by Mann–Whitney unpaired *U*-test. *p ≤ 0.05, **p ≤ 0.005, ***p ≤ 0.0005, and “n.s.” indicates not significant when compared to the media-treated group.

CD4 T cells that produce IFNγ (Th1), IL17 (Th17), or both IFNγ and IL17 (Th1 and Th17) play an important role in EAE by inducing inflammation and demyelination in the CNS (30, 31). Therefore, we compared the frequency of IFNγ- and IL17-producing CD4 T cells among CNS-infiltrating cells in the brain and spinal cord of mice that were induced with EAE by immunization with PLP_91−110_ and treated with *P. histicola* alone, Copaxone® alone, the combination of *P. histicola* and Copaxone®, or media alone. Mononuclear cells were isolated from the brain and spinal cord of mice and stimulated with the PLP_91−110_ peptide plus Brefeldin A for 14 h (32). Mice treated with *P. histicola* had a lower frequency of both IL17^+^CD4^+^ T cells and IFNγ^+^CD4^+^ T cells compared to those treated with media alone (IL17^+^CD4^+^ T cells, *P. histicola* vs. media: 1.89 ± 0.68 vs. 4.7 ± 1.55, *p* < 0.001; IFNγ^+^CD4^+^ T cells, *P. histicola* vs. media: 6.1 ± 2.3 vs. 9.8 ±1.84, *p* > 0.01) (Figures 5A,B). In addition, mice treated with *P. histicola* had lower levels of CD4 T cells expressing both IL17 and IFNγ (Figure 5D). These changes were not seen with Copaxone®. Notably, mice treated with Copaxone® did not show lower levels of IL17^+^CD4^+^ T cells or IFNγ^+^CD4^+^ T cells (Figures 5A–C). Similar to mice treated with *P. histicola* alone, the combination of both *P. histicola* and Copaxone® decreased the frequency of IFNγ^+^CD4^+^ T and IL17^+^CD4^+^ T cells (Figures 5A–C). We also analyzed levels of IL-17 producing CD4^+^ T cells in GALT to determine whether *P. histicola* or Copaxone® suppressed disease through modulation of gut resident IL17^+^CD4^+^ T cells. We did not observe any difference in IL-17 producing CD4^+^ T cells among different groups (Supplementary Figure 1). Thus, our data suggest that treatment with *P. histicola* alone or in combination with Copaxone® decreases the frequency of IFNγ^+^, IL17^+^ or both IFNγ plus IL17 producing CD4 T cells in the CNS of mice with EAE.

### *P. histicola* but Not Copaxone® Treatment Causes Restoration of Gut Microbiota

**Figure 6:**
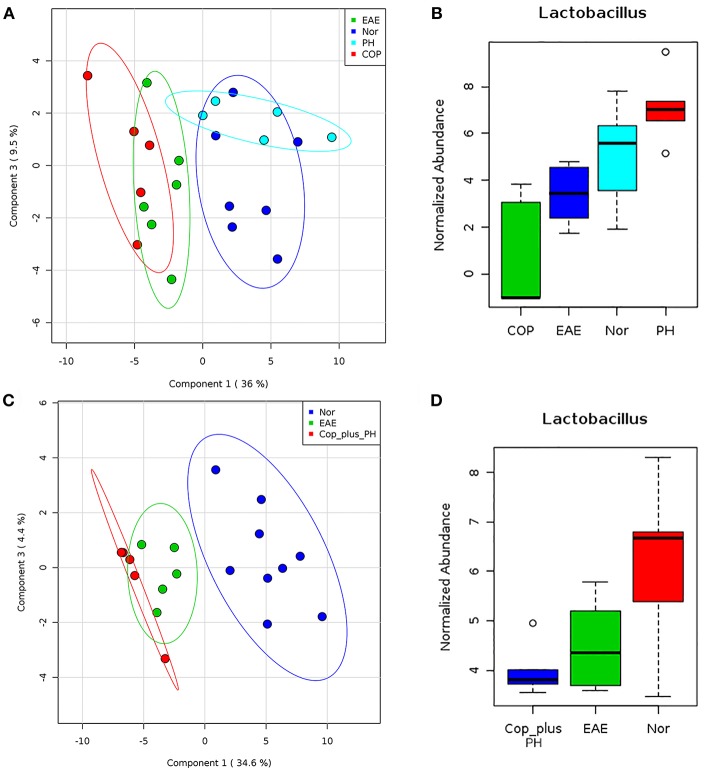
*P. histicola* but not Copaxone® mediates disease suppression through restoration of gut microbiota **(A)** Fecal samples were collected from pre-immunized HLA-DR3.DQ8 transgenic mice (Naïve/Nor), or mice immunized for EAE (EAE) and treated with Copaxone® (Cop) or *P. histicola* (PH). Fecal DNA was extracted, 16s rRNA (V3-V5) region was amplified, and sequenced on Illumina MiSeq platform. Two dimension Partial least square- dimension analysis (PLS-DA) scores plot of fecal microbiota from different treatment groups and naïve (Nor) mice with each dot representing a mice. **(B)** Box plot showing normalized relative abundance of *Lactobacillus* among different groups. **(C)** PLS-DA scores plot showing fecal microbiota profile from pre-immunized mice (Nor) or mice immunized for EAE (EAE) or Copaxone® plus *P. histicola* (Cop_plus_PH). **(D)** Box plot showing normalized relative abundance of *Lactobacillus* among different groups. Difference among groups were analyzed using one-way ANOVA (Kruskal–Wallis rank sum test) and FDR-adjusted *p* < 0.05.

We have previously shown that *P. histicola* can mediate its protective effect through modulation of gut microbiota (14). Therefore, we first investigated whether Copaxone® treatment can also modulate gut microbiota. Fecal microbiota analysis showed that development of EAE led to a shift in microbiota profile compared to naïve mice but the group treated with *P. histicola* showed a gut microbiota profile similar to naïve mice than those with EAE (Figure 6A). Although treatment with Copaxone® alone cause shift in gut microbiota, it was more similar to media treated EAE group than naïve mice (Figure 6A). Naïve mice showed higher relative abundance of *Lactobacillus* and *P. histicola* treated group also showed higher relative abundance of *Lactobacillus* (Figure 6B). However, media treated EAE group showed relative loss of bacteria belonging to *Lactobacillus* genera. Interestingly Copaxone® treated group also showed relative loss of *Lactobacillus* suggesting that Copaxone® might have different mechanism than *P. histicola* in regard to disease suppression. Finally we asked whether Copaxone® plus *P. histicola* treated group show gut microbiota profile similar to Copaxone® or *P. histicola* or different than both. As shown in Figure 6C, the combination of Copaxone® plus *P. histicola* treated group clustered closer to media treated EAE group than naïve mice. The combination group also showed lower levels of *Lactobacillus* compared to naïve mice (Figure 6D). Our gut microbiota profiling data suggests that the combination group behaved similar to Copaxone® alone group as they clustered together with media treated group characterized by relative loss of *Lactobacillus*. Thus, our data suggests that *P. histicola* might mediate its protective effect through restoration of gut microbiota to pre-immunized state whereas Copaxone® alone or combination of Copaxone® plus *P. histicola* might mediate their disease protective effect independent of gut microbiota.

## Discussion

Here, we compared the abilities of *P. histicola* or Copaxone® to suppress EAE in the HLA-DR3.DQ8 transgenic mouse model of MS, and identify potential mechanisms by which these treatments modulate disease. Using a preclinical model of MS, we report that *P. histicola* treatment suppresses EAE as efficiently as treatment with Copaxone®, but that the combination of *P. histicola* and Copaxone® is not more effective than either treatment alone. Treatment with *P. histicola* alone or *P. histicola* plus Copaxone® increased the frequency and number of CD4^+^ FoxP3^+^ regulatory T cells in the spleen as well as GALT and decreased the frequency of pro-inflammatory cytokine-producing CD4 T cells in the CNS of HLA-DR3.DQ8 transgenic mice that were induced with EAE. Thus, our results provide additional evidence that certain human gut commensal bacteria play an important role in ameliorating disease.

The human microbiome project (HMP) is an NIH initiative to catalog the human microbiome (33, 34), and has thus far identified an important role for the microbiota in human health and disease (35). Consistent with this, we and others have shown that there is enrichment and/or depletion of certain gut bacteria in patients with MS compared to HC, indicating that the gut microbiota plays an important role in disease pathogenesis (7–11). This suggests that specific human gut bacteria that are depleted or found in lower abundance in MS patients may have the potential to be used in the treatment of MS. Data from our group and others showed that the *Prevotella* genus is depleted in MS patients (7–9, 12) and that treatment with the specific strain, *P. histicola*, suppresses disease in preclinical animal models of MS (14) and rheumatoid arthritis (36). Various studies of other single bacteria stains such as *Bacteroides fragilis* (37–39), *Enterococcus faecium* strain L-3 (40), *Pediococcus acidilactici* (41), and a mixture of *Lactobacillus* strains (42) have also shown efficacy in suppressing CNS-specific disease in animal models (EAE) of MS. Thus, gut commensals offer an exciting new therapeutic avenue for the MS treatment.

In MS, autoreactive CD4 T cells that are activated in the periphery, traffic to the CNS and initiate an inflammatory cascade that results in demyelination and neuronal injury. In our mouse model of MS, we observed that treatment with *P. histicola* alone, Copaxone® alone, or the combination of *P. histicola* plus Copaxone® resulted in milder pathology in the brain and spinal cord indicating that these treatments suppressed either the infiltration and/or proliferation of inflammatory cells into the CNS, thus reducing inflammation and demyelination. Although Copaxone® treated group showed few inflammatory cells in the brain tissue, it is possible that these cells are regulatory in nature. Previous studies have shown that Copaxone® can mediate its disease protective effects through induction of regulatory myeloid or CD8^+^ T cells (21, 43). The milder pathology in the brain and spinal cord of Copaxone®-treated- mice is supported by previous study (44). Our data suggests that *P. histicola* is as effective as Copaxone® at reducing the pathology associated with disease.

MS is an inflammatory disease in which the balance between pro-inflammatory Th1/Th17 cells and anti-inflammatory CD4^+^FoxP3^+^ regulatory T cells is shifted toward a pro-inflammatory response. Therefore, potential therapeutic agents could act to suppress disease by restoring this balance toward an anti-inflammatory response, either by inducing Tregs, suppressing pro-inflammatory Th1/Th17 cells, or affecting both cell types in the periphery and in the CNS (14, 37, 38, 40–42, 45). The disease suppressive effects of *B. fragilis* and *Lactobacillus species* (mixture) in EAE were mediated by inducing CD4^+^FoxP3^+^ regulatory T cells and production of IL10 (42, 46). Similarly, we found that disease suppression mediated by *P. histicola* was associated with a higher frequency and number of CD4^+^FoxP3^+^ regulatory T cells in the gut as well as periphery. Our earlier studies also demonstrated that *P. histicola* induced CD4^+^FoxP3^+^ regulatory T cells and that these cells had higher suppressive capabilities (14). These results suggest that *P. histicola* can mediates disease suppression through induction of CD4^+^FoxP3^+^ regulatory T cells. The mechanism through which *P. histicola* induce CD4^+^FoxP3^+^ regulatory T cells is not well-understood. We hypothesize that *P. histicola* can induce Tregs through its ability to produce secondary metabolite such as short chain fatty acid (SCFA) and phytoestrogen metabolites (47). SCFA such as acetate and butyrate had been shown to induce CD4^+^FoxP3^+^ regulatory T cells (48). Certain beneficial bacteria such as *B. fragilis* can mediate their effect through capsular polysacchride A (PSA) (39, 46, 49), however at present we do not know whether similar mechanism is true for *P. histicola*.

Collective evidence suggests that IL17- and IFNγ-producing CD4 T cells are the major pro-inflammatory cells involved in the pathogenesis of both MS and the EAE (30, 50, 51). Treatment with *P. histicola* alone or in combination with Copaxone® decreased the percentage of IL17^+^ and IFNγ^+^ CD4^+^ T cells infiltrating the CNS of HLA-DR3.DQ8 transgenic mice. Interestingly, treatment with *P. histicola* alone also reduced infiltration of CD4 T cells that are positive for both IFNγ and IL17 (IFNγ^+^IL17^+^ CD4 T cells). Recent studies suggest that IFNγ^+^IL17^+^ CD4 T cells from humans are more pathogenic than CD4 T cells that are only producing one cytokine, i.e., either IFNγ^+^ or IL17^+^ (52). Thus, our data indicate that *P. histicola* alone or in combination with Copaxone® suppresses inflammation and demyelination by suppressing pro-inflammatory Th1 and Th17 responses. In our study, mice treated with Copaxone® did not show reduced levels of CD4 T cells expressing either IL17 or IFNγ or those expressing both IFNγ^+^IL17^+^ in the CNS. Although the exact mechanism by which Copaxone® suppress disease is not well-understood, previous studies have suggested it acts by diverting the immune response from a pro-inflammatory Th1 phenotype toward that of a Th2 phenotype in which cytokines such as IL5 are produced (16). Additionally, Copaxone®-induced EAE disease resistance in SJL mice is associated with the presence of Copaxone®-specific Th2 cells in the CNS and, upon adoptive transfer, these cells can be detected in the CNS of recipient mice (44). Copaxone® is considered to be an altered peptide ligand (APL) which was originally developed as an MBP mimic of myelin basic protein, a major constituent of myelin sheath. However, due to its ability to bind strongly to the MS-linked HLA-DRB1^*^1501 allele and induce a tolerogenic Th2 response, Copaxone® can act as a degenerate T cell antigen. Our data indicates that treatment with Copaxone® fails to alter the frequency of pathogenic Th1 and Th17 cells, whereas this population is directly affected upon administration of *P. histicola*. Failure of Copaxone® to suppress the frequency of pathogenic Th1 and Th17 cells indicate that *P. histicola* and Copaxone® might have different mechanism of action.

Ability of *P. histicola* to restore gut microbiota in EAE model, are in line with our previous finding where treatment with *P. histicola* resulted in restoration of gut microbiota to a preimmunized state (14). Interestingly, Copaxone® treated EAE group has gut microbiota similar to media treated EAE group suggesting that Copaxone® mediated disease suppression are either independent of gut microbiota or different than *P. histicola* mediated effect. Although Jangi et al. (9) have shown that MS patients on disease modifying therapies such as Copaxone® and IFNβ-1b had higher levels of *Prevotella*, it is possible that the change in *Prevotella* levels were due to IFNβ-1b and not specifically due to Copaxone®. This argument is supported by two recent studies showing that IFNβ-1b treated MS patients have higher levels of *Prevotella* (53) and MS patients receiving Copaxone® did not show any change in levels of *Prevotella* (54, 55). Thus, our gut microbiota data also points toward a non-overlapping effect of *P. histicola* and Copaxone® in regard to disease suppression in EAE model.

Our result indicates that *P. histicola* and Copaxone® have both overlapping and non-overlapping modes of action in HLA-DR3.DQ8 transgenic mice. We found that *P. histicola* alone or in combination with Copaxone® induces regulatory T cells in the spleen and suppresses inflammatory cytokine-producing CD4 T cells in the CNS of HLA-DR3.DQ8 transgenic mice. Whereas, treatment with Copaxone® suppressed disease in these mice, this was neither due to induction of CD4^+^FoxP3^+^ regulatory T cells nor due to suppression of inflammatory cytokine-producing CD4 T cells.

In summary, our findings indicate that monotherapy with *P. histicola* suppresses EAE in HLA-DR3.DQ8 transgenic mice as efficiently as Copaxone® and provides additional evidence that the gut bacteria can be used as therapeutic agents to ameliorate autoimmune inflammatory diseases such as MS. Specifically, our study suggests the possibility of using *P. histicola* in the development of a novel effective therapy for MS and other neuroinflammatory diseases.

## Author Contributions

AKM conceptualized the study, designed, and performed the experiments, wrote the manuscript, and gave final approval of the manuscript to be published. SS designed and performed the experiments, analyses the data, and helped with writing the manuscript. SF helped with experimental design and performing experiment. ACM performed mouse genotyping and microbiome DNA extraction. KZ and RS performed microbiome analysis. KG-C performed brain and spinal cord pathology. JM and NK helped with the study design and interpretation of the data. All authors commented on the manuscript.

### Conflict of Interest Statement

AKM and JM are inventors of the use of *Prevotella histicola* for treatment of autoimmune disease, used in this study and the patent is owned by Mayo Clinic Rochester, USA. The technology has been licensed by Mayo Clinic to Evelo Biosciences. AKM and JM received royalties from Mayo Clinic (paid by Evelo Biosciences). The remaining authors declare that the research was conducted in the absence of any commercial or financial relationships that could be construed as a potential conflict of interest.

## References

[1] Stinissen P Raus J Zhang J. Autoimmune pathogenesis of multiple sclerosis: role of autoreactive T lymphocytes and new immunotherapeutic strategies. Crit Rev Immunol. (1997) 17:33–75. 10.1615/CritRevImmunol.v17.i1.20 9034723

[2] Willer CJ Dyment DA Risch NJ Sadovnick AD Ebers GC Canadian Collaborative Study G. Twin concordance and sibling recurrence rates in multiple sclerosis. Proc Natl Acad Sci USA. (2003) 100:12877–82. 10.1073/pnas.1932604100 14569025 PMC240712

[3] Hollenbach JA Oksenberg JR. The immunogenetics of multiple sclerosis: a comprehensive review. J Autoimmun. (2015) 64:13–25. 10.1016/j.jaut.2015.06.010 26142251 PMC4687745

[4] Goodin DS. The causal cascade to multiple sclerosis: a model for MS pathogenesis. PLoS ONE. (2009) 4:e4565. 10.1371/journal.pone.0004565 19242548 PMC2644781

[5] Ramagopalan SV Handel AE Giovannoni G Rutherford Siegel S Ebers GC Chaplin G. Relationship of UV exposure to prevalence of multiple sclerosis in England. Neurology. (2011) 76:1410–4. 10.1212/WNL.0b013e318216715e 21502600 PMC3087404

[6] Ochoa-Reparaz J Kasper LH. Gut microbiome and the risk factors in central nervous system autoimmunity. FEBS Lett. (2014) 588:4214–22. 10.1016/j.febslet.2014.09.024 25286403 PMC4254300

[7] Miyake S Kim S Suda W Oshima K Nakamura M Matsuoka T. Dysbiosis in the gut microbiota of patients with multiple sclerosis, with a striking depletion of species belonging to clostridia XIVa and IV clusters. PLoS ONE. (2015) 10:e0137429. 10.1371/journal.pone.0137429 26367776 PMC4569432

[8] Chen J Chia N Kalari KR Yao JZ Novotna M Paz Soldan MM. Multiple sclerosis patients have a distinct gut microbiota compared to healthy controls. Sci Rep. (2016) 6:28484. 10.1038/srep28484 27346372 PMC4921909

[9] Jangi S Gandhi R Cox LM Li N Von Glehn F Yan R. Alterations of the human gut microbiome in multiple sclerosis. Nat Commun. (2016) 7:12015. 10.1038/ncomms12015 27352007 PMC4931233

[10] Tremlett H Fadrosh DW Faruqi AA Hart J Roalstad S Graves J. Gut microbiota composition and relapse risk in pediatric MS: a pilot study. J Neurol Sci. (2016) 363:153–7. 10.1016/j.jns.2016.02.042 27000242 PMC4806409

[11] Colpitts SL Kasper EJ Keever A Liljenberg C Kirby T Magori K. A bidirectional association between the gut microbiota and CNS disease in a biphasic murine model of multiple sclerosis. Gut Microbes. (2017) 8:561–73. 10.1080/19490976.2017.1353843 28708466 PMC5730387

[12] Cosorich I Dalla-Costa G Sorini C Ferrarese R Messina MJ Dolpady J. High frequency of intestinal TH17 cells correlates with microbiota alterations and disease activity in multiple sclerosis. Sci Adv. (2017) 3:e1700492. 10.1126/sciadv.1700492 28706993 PMC5507635

[13] Mangalam A Luckey D Basal E Jackson M Smart M Rodriguez M. HLA-DQ8 (DQB1^*^0302)-restricted Th17 cells exacerbate experimental autoimmune encephalomyelitis in HLA-DR3-transgenic mice. J Immunol. (2009) 182:5131–9. 10.4049/jimmunol.0803918 19342694 PMC2665933

[14] Mangalam A Shahi SK Luckey D Karau M Marietta E Luo N. Human gut-derived commensal bacteria suppress CNS inflammatory and demyelinating disease. Cell Rep. (2017) 20:1269–77. 10.1016/j.celrep.2017.07.031 28793252 PMC5763484

[15] Teitelbaum D Aharoni R Arnon R Sela M. Specific inhibition of the T-cell response to myelin basic protein by the synthetic copolymer Cop 1. Proc Natl Acad Sci USA. (1988) 85:9724–8. 10.1073/pnas.85.24.9724 2462252 PMC282850

[16] Duda PW Schmied MC Cook SL Krieger JI Hafler DA. Glatiramer acetate (Copaxone) induces degenerate, Th2-polarized immune responses in patients with multiple sclerosis. J Clin Invest. (2000) 105:967–76. 10.1172/JCI8970 10749576 PMC377485

[17] Neuhaus O Farina C Yassouridis A Wiendl H Then Bergh F Dose T. Multiple sclerosis: comparison of copolymer-1- reactive T cell lines from treated and untreated subjects reveals cytokine shift from T helper 1 to T helper 2 cells. Proc Natl Acad Sci USA. (2000) 97:7452–7. 10.1073/pnas.97.13.7452 10861011 PMC16566

[18] Polman CH Uitdehaag BM. New and emerging treatment options for multiple sclerosis. Lancet Neurol. (2003) 2:563–6. 10.1016/S1474-4422(03)00505-2 12941579

[19] Stuve O Youssef S Dunn S Slavin AJ Steinman L Zamvil SS. The potential therapeutic role of statins in central nervous system autoimmune disorders. Cell Mol Life Sci. (2003) 60:2483–91. 10.1007/s00018-003-3146-0 14625690 PMC11138936

[20] Das P Drescher KM Geluk A Bradley DS Rodriguez M David CS. Complementation between specific HLA-DR and HLA-DQ genes in transgenic mice determines susceptibility to experimental autoimmune encephalomyelitis. Hum Immunol. (2000) 61:279–89. 10.1016/S0198-8859(99)00135-4 10689117

[21] Tyler AF Mendoza JP Firan M Karandikar NJ. CD8(+) T cells are required for glatiramer acetate therapy in autoimmune demyelinating disease. PLoS ONE. (2013) 8:e66772. 10.1371/journal.pone.0066772 23805274 PMC3689655

[22] Teitelbaum D Arnon R Sela M. Immunomodulation of experimental autoimmune encephalomyelitis by oral administration of copolymer 1. Proc Natl Acad Sci USA. (1999) 96:3842–7. 10.1073/pnas.96.7.3842 10097125 PMC22382

[23] Mangalam AK Luo N Luckey D Papke L Hubbard A Wussow A. Absence of IFN-gamma increases brain pathology in experimental autoimmune encephalomyelitis-susceptible DRB1^*^0301.DQ8 HLA transgenic mice through secretion of proinflammatory cytokine IL-17 and induction of pathogenic monocytes/microglia into the central nervous system. J Immunol. (2014) 193:4859–4870. 10.4049/jimmunol.1302008 25339670 PMC4233133

[24] Pino PA Cardona AE. Isolation of brain and spinal cord mononuclear cells using percoll gradients. J Vis Exp. (2011) 2348. 10.3791/2348 21339713 PMC3339837

[25] Couter CJ Surana NK. Isolation and flow cytometric characterization of murine small intestinal lymphocytes. J Vis Exp. (2016). 10.3791/54114 27213538 PMC4942069

[26] Zhang J Kobert K Flouri T Stamatakis A. PEAR: a fast and accurate Illumina Paired-End reAd mergeR. Bioinformatics. (2014) 30:614–20. 10.1093/bioinformatics/btt593 24142950 PMC3933873

[27] Angiuoli SV Matalka M Gussman A Galens K Vangala M Riley DR. CloVR: a virtual machine for automated and portable sequence analysis from the desktop using cloud computing. BMC Bioinformatics. (2011) 12:356. 10.1186/1471-2105-12-356 21878105 PMC3228541

[28] Arndt D Xia J Liu Y Zhou Y Guo AC Cruz JA. METAGENassist: a comprehensive web server for comparative metagenomics. Nucleic Acids Res. (2012) 40:W88–95. 10.1093/nar/gks497 22645318 PMC3394294

[29] O'connor RA Anderton SM. Foxp3+ regulatory T cells in the control of experimental CNS autoimmune disease. J Neuroimmunol. (2008) 193:1–11. 10.1016/j.jneuroim.2007.11.016 18077005

[30] O'Connor RA Prendergast CT Sabatos CA Lau CW Leech MD Wraith DC. Cutting edge: Th1 cells facilitate the entry of Th17 cells to the central nervous system during experimental autoimmune encephalomyelitis. J Immunol. (2008) 181:3750–4. 10.4049/jimmunol.181.6.3750 18768826 PMC2619513

[31] Domingues HS Mues M Lassmann H Wekerle H Krishnamoorthy G. Functional and pathogenic differences of Th1 and Th17 cells in experimental autoimmune encephalomyelitis. PLoS ONE. (2010) 5:e15531. 10.1371/journal.pone.0015531 21209700 PMC3000428

[32] Itani FR Sinha S Brate AA Pewe LL Gibson-Corley KN Harty JT. Suppression of autoimmune demyelinating disease by preferential stimulation of CNS-specific CD8 T cells using Listeria-encoded neuroantigen. Sci Rep. (2017) 7:1519. 10.1038/s41598-017-01771-8 28484224 PMC5431563

[33] Human Microbiome Project C A framework for human microbiome research. Nature. (2012) 486:215–21. 10.1038/nature11209 22699610 PMC3377744

[34] O'shea JJ Jones RG. Autoimmunity: Rubbing salt in the wound. Nature. (2013) 496:437–439. 10.1038/nature11959 23467087

[35] Cho I Blaser MJ. The human microbiome: at the interface of health and disease. Nat Rev Genet. (2012) 13:260–70. 10.1038/nrg3182 22411464 PMC3418802

[36] Marietta EV Murray JA Luckey DH Jeraldo PR Lamba A Patel R. Suppression of inflammatory arthritis by human gut-derived prevotella histicola in humanized mice. Arthritis Rheumatol. (2016) 68:2878–88. 10.1002/art.39785 27337150 PMC5125894

[37] Round JL Mazmanian SK. Inducible Foxp3+ regulatory T-cell development by a commensal bacterium of the intestinal microbiota. Proc Natl Acad Sci USA. (2010) 107:12204–9. 10.1073/pnas.0909122107 20566854 PMC2901479

[38] Surana NK Kasper DL. The yin yang of bacterial polysaccharides: lessons learned from B. fragilis PSA Immunol Rev. (2012) 245:13–26. 10.1111/j.1600-065X.2011.01075.x 22168411 PMC3243960

[39] Wang Y Telesford KM Ochoa-Reparaz J Haque-Begum S Christy M Kasper EJ. An intestinal commensal symbiosis factor controls neuroinflammation via TLR2-mediated CD39 signalling. Nat Commun. (2014) 5:4432. 10.1038/ncomms5432 25043484 PMC4118494

[40] Abdurasulova IN Matsulevich AV Tarasova EA Kudryavtsev IV Serebrjakova MK Ermolenko EI. Enterococcus faecium strain L-3 and glatiramer acetate ameliorate experimental allergic encephalomyelitis in rats by affecting different populations of immune cells. Benef Microbes. (2016) 7:719–29. 10.3920/BM2016.0018 27633171

[41] Takata K Kinoshita M Okuno T Moriya M Kohda T Honorat JA. The lactic acid bacterium *Pediococcus acidilactici* suppresses autoimmune encephalomyelitis by inducing IL-10-producing regulatory T cells. PLoS ONE. (2011) 6:e27644. 10.1371/journal.pone.0027644 22110705 PMC3217013

[42] Lavasani S Dzhambazov B Nouri M Fak F Buske S Molin G. A novel probiotic mixture exerts a therapeutic effect on experimental autoimmune encephalomyelitis mediated by IL-10 producing regulatory T cells. PLoS ONE. (2010) 5:e9009. 10.1371/journal.pone.0009009 20126401 PMC2814855

[43] Prod'homme T Zamvil SS. The evolving mechanisms of action of glatiramer acetate. Cold Spring Harb Perspect Med. (2019) 9:a029249. 10.1101/cshperspect.a029249 29440323 PMC6360864

[44] Aharoni R Teitelbaum D Leitner O Meshorer A Sela M Arnon R. Specific Th2 cells accumulate in the central nervous system of mice protected against experimental autoimmune encephalomyelitis by copolymer 1. Proc Natl Acad Sci USA. (2000) 97:11472–7. 10.1073/pnas.97.21.11472 11027347 PMC17224

[45] Wender M Michalak S Wygladalska-Jernas H. The effect of short-term treatment with interferon beta 1a on acute experimental allergic encephalomyelitis. Folia Neuropathol. (2001) 39:91–3. 10.1007/s00005-017-0458-6 11680640

[46] Ochoa-Reparaz J Mielcarz DW Wang Y Begum-Haque S Dasgupta S Kasper DL. A polysaccharide from the human commensal Bacteroides fragilis protects against CNS demyelinating disease. Mucosal Immunol. (2010) 3:487–95. 10.1038/mi.2010.29 20531465

[47] Freedman SN Shahi SK Mangalam AK. The “Gut Feeling”: breaking down the role of gut microbiome in multiple sclerosis. Neurotherapeutics. (2018) 15:109–25. 10.1007/s13311-017-0588-x 29204955 PMC5794701

[48] Zeng H Chi H. Metabolic control of regulatory T cell development and function. Trends Immunol. (2015) 36:3–12. 10.1016/j.it.2014.08.003 25248463 PMC4280284

[49] Troy EB Kasper DL. Beneficial effects of Bacteroides fragilis polysaccharides on the immune system. Front Biosci. (2010) 15:25–34. 10.2741/3603 20036803 PMC2995369

[50] Mcfarland HF Martin R. Multiple sclerosis: a complicated picture of autoimmunity. Nat Immunol. (2007) 8:913–9. 10.1038/ni1507 17712344

[51] Tzartos JS Friese MA Craner MJ Palace J Newcombe J Esiri MM. Interleukin-17 production in central nervous system-infiltrating T cells and glial cells is associated with active disease in multiple sclerosis. Am J Pathol. (2008) 172:146–55. 10.2353/ajpath.2008.070690 18156204 PMC2189615

[52] Hu D Notarbartolo S Croonenborghs T Patel B Cialic R Yang TH. Transcriptional signature of human pro-inflammatory TH17 cells identifies reduced IL10 gene expression in multiple sclerosis. Nat Commun. (2017) 8:1600. 10.1038/s41467-017-01571-8 29150604 PMC5693957

[53] Castillo-Alvarez F Perez-Matute P Oteo JA Marzo-Sola ME. The influence of interferon beta-1b on gut microbiota composition in patients with multiple sclerosis. Neurologia. (2018). 10.1016/j.nrl.2018.04.006. [Epub ahead of print]. 34537163

[54] Katz Sand I Zhu Y Ntranos A Clemente JC Cekanaviciute E Brandstadter R. Disease-modifying therapies alter gut microbial composition in MS. Neurol Neuroimmunol Neuroinflamm. (2019) 6:e517. 10.1212/NXI.0000000000000517 30568995 PMC6278850

[55] Mangalam AK. Drugs, bugs, and MS: the interplay between disease-modifying therapy and gut microbiota. Neurol Neuroimmunol Neuroinflamm. (2019) 6:e524. 10.1212/NXI.0000000000000524 30569001 PMC6278853

