# Supplementary material for: Prevotella histicola, A Human Gut Commensal, Is as Potent as COPAXONE® in an Animal Model of Multiple Sclerosis
Source: Front Immunol. 2019 Mar 22;10:462. doi: 10.3389/fimmu.2019.00462 (PMC6448018; doi:10.3389/fimmu.2019.00462)
Supplement: Supplementary file 1 [file Presentation_1.PPTX]

## Slide 1
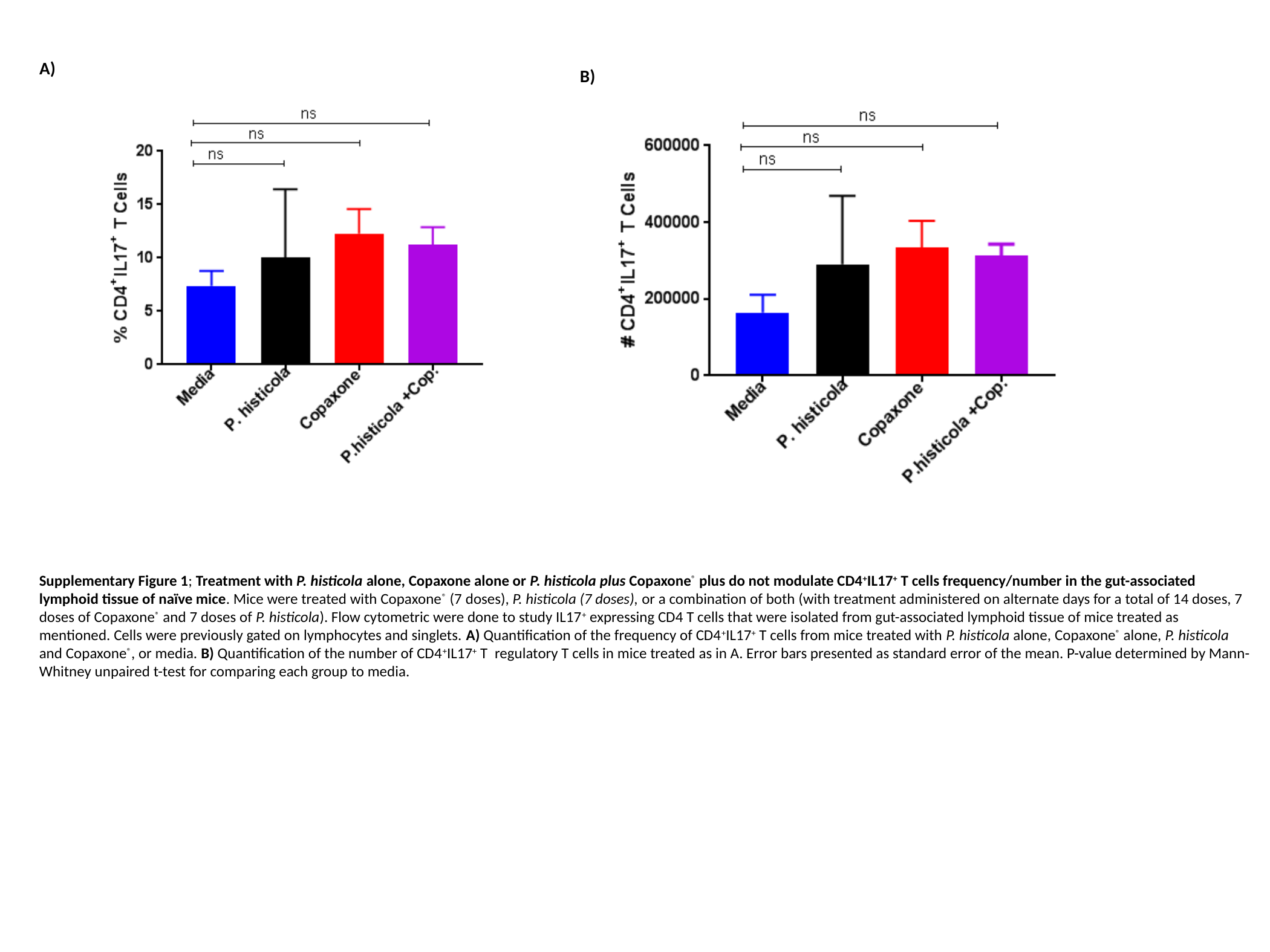

A)
B)
Supplementary Figure 1; Treatment with P. histicola alone, Copaxone alone or P. histicola plus Copaxone® plus do not modulate CD4+IL17+ T cells frequency/number in the gut-associated lymphoid tissue of naïve mice. Mice were treated with Copaxone® (7 doses), P. histicola (7 doses), or a combination of both (with treatment administered on alternate days for a total of 14 doses, 7 doses of Copaxone® and 7 doses of P. histicola). Flow cytometric were done to study IL17+ expressing CD4 T cells that were isolated from gut-associated lymphoid tissue of mice treated as mentioned. Cells were previously gated on lymphocytes and singlets. A) Quantification of the frequency of CD4+IL17+ T cells from mice treated with P. histicola alone, Copaxone® alone, P. histicola and Copaxone®, or media. B) Quantification of the number of CD4+IL17+ T regulatory T cells in mice treated as in A. Error bars presented as standard error of the mean. P-value determined by Mann- Whitney unpaired t-test for comparing each group to media.
